# Antimicrobial efficacy of chlorhexidine-treated surfaces against clinical isolates implicated in nosocomial infections

**DOI:** 10.1099/jmm.0.002025

**Published:** 2025-06-24

**Keywords:** antimicrobial resistance, antimicrobial surfaces, chlorhexidine, clinical isolates

## Abstract

**Introduction.** Bacterial infections and antimicrobial resistance are significant threats to global public health, both of which spread through contamination of solid surfaces. We have previously developed an antimicrobial surface technology that directly bonds the broad-spectrum biocide chlorhexidine to steel surfaces. These surfaces were shown to kill bacteria within minutes of contact and to be effective against bacteria evolved in the laboratory for resistance to chlorhexidine in solution.

**Hypothesis/Gap Statement.** We hypothesized that resistance to these surfaces could exist outside of the naive and chlorhexidine-resistant laboratory strains tested previously. We also sought to test whether strains that were resistant to chlorhexidine in solution were also resistant to chlorhexidine-based antimicrobial surfaces.

**Aim.** To test the efficacy of these surfaces against a range of bacteria isolated from the hospital environment and to compare this to the resistance of these bacteria to chlorhexidine in solution or when dissolved in solid media.

**Methodology.** Ninety-one isolates of mixed bacterial species were obtained from Queen Elizabeth Hospital Birmingham. The isolates, along with laboratory strains of *Escherichia coli*, *Pseudomonas aeruginosa* and *Staphylococcus aureus*, were tested for sensitivity to chlorhexidine-coated steel surfaces in a 30-min exposure simulated splash assay. Resistance to chlorhexidine in solution was also assayed by solid and broth media MIC assays.

**Results.** We demonstrate that within 30 min of incubation, the surfaces reduced the survival of all 91 isolates. Over 85% of these isolates were killed (exhibiting a 7–8 log reduction compared with control surfaces), whilst 12% experienced a 3–4 log reduction. We also show that resistance to the surfaces did not correlate with resistance to freely diffusible chlorhexidine in liquid or solid media.

**Conclusion.** The results demonstrate the efficacy of chlorhexidine-coated surfaces against a broad range of bacterial isolates from the hospital environment and imply the potential for a mode of exposure to dictate the effectiveness of different antimicrobial resistance mechanisms. Future studies should investigate the genetic mechanisms providing resistance to chlorhexidine-coated surfaces and whether these differ in the capacity to provide resistance to chlorhexidine in different modes of exposure.

**Impact Statement**

Surfaces represent a significant source of bacterial contamination that can lead to the spread of these organisms and potentially infection, especially in high-risk environments such as hospitals. Surfaces also provide an environment for bacteria to interact and spread mechanisms of resistance to the antibiotics that are used to combat infection. To address this, we previously developed a modified steel surface with antimicrobial properties that were highly robust and capable of killing bacteria, viruses and fungi within minutes of them landing on the surface. Here, we tested whether these surfaces were active against a range of different bacteria isolated from the hospital environment to understand if these observations apply to the bacteria for which the surfaces were designed. We show that very few isolates were able to survive on the surfaces and even these were reduced in their capacity to survive the surfaces. We also show that resistance of the bacteria to the antimicrobial included in the surface did not align with whether they were resistant to the surfaces. This work paves the way for future studies on which antimicrobial resistance mechanisms are functional against the antimicrobial when incorporated in different formulations. This will allow us to understand how exposure to antimicrobials can be modified to potentially circumvent resistance mechanisms.

## Data Summary

The authors confirm that all supporting data, code and protocols have been provided within the article or through supplementary data files.

## Introduction

Infections caused by bacteria and antimicrobial resistance are a significant and serious threat to global public health, with an estimated 7.7 million deaths annually being attributed to infections caused by 33 bacterial species. This makes them a leading cause of infectious mortality and accounts for ~11% of all deaths worldwide [1,2]. Bacterial diseases contribute significantly to global health burdens, especially in low-income regions, and antimicrobial resistance is a growing concern contributing to nearly 5 million deaths annually. Compared with other major causes of death, bacterial infections remain a critical public health concern, particularly due to rising antibiotic resistance and hospital-acquired infections [2].

Healthcare-associated infections (HAIs) pose a significant challenge to patient safety, leading to increased morbidity, mortality and healthcare costs [3,4]. Effective infection control measures are critical in mitigating the transmission of pathogens in hospital settings. These measures include hand hygiene, sterilization of medical instruments, use of personal protective equipment, environmental cleaning and advanced disinfection technologies. Several disinfection strategies have been developed and assessed for their effectiveness in reducing microbial contamination in healthcare environments including chemical disinfection, UV light disinfection and aerosolized disinfection methods such as hydrogen peroxide vapour systems [5]. Recent literature review studies on infection control on hospital surfaces, such as medical instruments, have highlighted that using hydrogen peroxide (H_2_O_2_) as a disinfectant through automated disinfection systems was particularly effective [6]. In addition, chlorine-based disinfectants, such as sodium dichloroisocyanurate and chloroxylenol, are shown to have good efficacy against bacterial type strains and their respective multi-drug-resistant hospital isolates [7]. However, these disinfection choices are limited in various applications such as hand hygiene practices, pre-surgical skin preparations and wound disinfections. Furthermore, the use of chlorine-based disinfectants requires strict adherence to the preparation, storage and use of freshly prepared solutions and frequent reapplication/disinfection.

Contaminated surfaces present a major route for the spread of bacterial infections, especially within hospital settings [8,9]. Surfaces also represent a potential environment for the exchange of genetic material that enables the spread of antimicrobial resistance amongst bacterial populations [10,11]. As such, modification of surfaces to have antimicrobial properties represents an excellent opportunity to prevent the spread of infection-causing bacteria and combat the rising rates of antimicrobial resistance. Antimicrobial surfaces (AMSs) utilize various mechanisms to destroy or inhibit the growth of micro-organisms upon contact. These mechanisms can be broadly categorized as (a) contact active killing by chemically modified surfaces (e.g. biocide or antimicrobial peptide bonded to surfaces), (b) leaching or releasing of active agents (zinc, copper and silver ions), (c) photocatalysis (titanium dioxide with UV) or (d) topographical modifications, each of which offers unique benefits depending on the type of coating or material used. The integration of AMSs into hospital settings has the potential to significantly enhance infection control measures [12,14].

There has been substantial progress in the development of antimicrobial materials for healthcare applications, such as using solid copper and triclosan-based elution coatings. Copper has been used as the preferred material of choice in healthcare settings for AMSs in recent years, appearing to exert its antimicrobial effects through multimodal action [15]. Developments in nanoparticle technology have also enabled copper nanoparticles to be impregnated into soft materials such as cotton that resist common washing cycles and should enable the development of antimicrobial bedsheets [16]. However, despite these successes, it typically takes several hours for copper to exert antimicrobial effects [17]. As a solution to this, the use of triclosan in surface coatings has been investigated. Triclosan is a broad-spectrum antibacterial and antifungal agent, which acts by inhibiting fatty acid synthesis, leading to membrane destabilization and cell death [18]. Triclosan has been incorporated into antimicrobial sutures, eluting triclosan locally at the surgical site to prevent infections. Whilst these sutures are highly effective at preventing surgical site infections, there are questions about the long-term use of triclosan as a surface coating due to it causing skin irritation, allergic vulnerability and destruction of fragile aquatic ecosystems [19]. Extensive use of triclosan as an antiseptic and disinfectant has led to the development of triclosan resistance; this is especially concerning as triclosan resistance can provide cross-resistance to other antibiotics and biocidal agents [20]. As such, there is a need to develop alternative AMS coating technologies that address these issues and are durable, have long-lasting efficacy, are cost-effective and can kill pathogenic micro-organisms in a short period of time.

A promising alternative broad-spectrum AMS agent that addresses many of the broader issues associated with AMS coatings is chlorhexidine digluconate (CHX). CHX is a widely used antiseptic agent known for its broad-spectrum antimicrobial properties and effectiveness against various pathogens including bacteria, viruses and fungi [21]. This antimicrobial agent is available in a range of formulations and is commonly applied to surfaces in healthcare settings, such as medical devices, catheters and surgical instruments, to create a protective barrier against pathogens, thus preventing various HAIs and promoting patient safety [3,22, 23]. Depending on the concentration, CHX has both bacteriostatic and bactericidal mechanisms of action [24]. CHX has a cationic molecular component that attaches to negatively charged bacterial cell membranes through interactions with molecules such as the phosphate groups in LPS as well as the carboxyl groups in the proteins [25]. At high concentrations, CHX acts as a detergent and causes complete precipitation of the cytoplasm, whereas at sublethal concentrations, CHX leads to increased permeability of the cell membrane [25]. How the incorporation of CHX into AMS technologies affects the mode of action remains to be investigated.

CHX AMS coatings have gained significant attention in recent years as a potential way to inhibit the growth of micro-organisms and reduce the risk of infections [26,29]. Other groups have demonstrated the ability to coat titanium [30] with CHX, and our group has developed methods for coating plastics [31] and air filters [32]. In addition, we have also previously demonstrated the capacity to chemically bond CHX, and other actives such as antimicrobial peptides, to steel surfaces. This prevents the release of CHX but maintains antimicrobial activity [27,33]. We demonstrated that nitriding the steel leads to surface activation, after which CHX is coupled to the surface through incubation with peptide bond coupling reagents. Whilst we have proven that the CHX is bonded to the surface by time-of-flight secondary ion MS [27], we can only hypothesize about the nature of the surface-tethered CHX molecule. Unlike unbound CHX, which acts as a bolaamphiphilic biscationic quaternary ammonium compound with two cationic groups for bacterial cell lysis, the tethered form could function differently. It is likely that one bisguanidinium residue anchors to the steel surface, whilst the other remains available for antimicrobial activity. The presence of dual cationic charges separated by a linker in the free form of CHX has been reported to provide distinct advantages for antimicrobial activity [34]. Consequently, a differential biological activity profile could be anticipated for this steel-tethered system.

The CHX-bonded steel AMS we previously developed displayed excellent antimicrobial efficacy against Gram-negative and Gram-positive model pathogenic bacteria as well as fungi and viruses [27]. These AMSs have been shown to kill these micro-organisms much faster than commercially available technologies. In addition, the method of coating CHX onto steel surfaces enables CHX to be permanently bonded to the surface and resistant to elution, which is essential in hospital settings where frequent cleaning regimens are important. The AMS polymer coatings have also been shown to be resistant to the elution of CHX through washing and to be effective against bacteria that had been evolved for resistance to CHX in solution within the laboratory [31]. Here, we expand this observation to analyse the efficacy of CHX AMS steel against a collection of clinical isolates representing a wide range of bacterial species from the hospital environment. We then compare the resistance of these isolates to CHX in solution, when dissolved in solid media or when permanently bonded to the AMS.

## Methods

All consumables were obtained from Sigma-Aldrich, UK, unless otherwise stated. Steel surfaces were obtained and nitrided commercially by Rubig GmbH, Austria. Zirconium oxide beads were purchased from Thistle Scientific Ltd, and the 12-well tissue culture plates were purchased from Corning, UK.

### Bacterial strains

Bacteria were routinely cultured in lysogeny broth (LB) at 37 °C. Laboratory stocks of bacterial strains used in this study for comparison against the clinical isolates were *Escherichia coli* BW25113, *E. coli* ATCC25922 and *Pseudomonas aeruginosa* PAO1. Clinical isolates were obtained from Queen Elizabeth Hospital (QEH) Birmingham, UK, following the identification and characterization by QEH Birmingham microbiology laboratory. Whilst all strains were confirmed, the identity of some was lost; therefore, they have been labelled as ‘unknown’. Clinical bacterial strains consisted of the most common bacterial species found within hospitals and were selected for comparative analysis.

### Coating of steel surfaces

Nitrided steel surfaces were cleaned using acetone and acetonitrile before being incubated in a coating mixture [acetonitrile, 52 mM 2-(1H-benzotriazol-1-yl)-1,1,3,3-tetramethyluronium hexafluorophosphate (Scientific Laboratory Supplies, Hessle, UK), 10% *N*,*N*-diisopropylethylamine and 0.33% CHX] 6 ml cm^−2^ of surface. Surfaces were coated with continuous agitation at room temperature (18–22 °C) for 16–18 h. Surfaces were then removed, washed with acetonitrile and acetone to remove unbound material, air dried and stored in the dark at room temperature (18–22 °C). Control surfaces were also washed with acetonitrile and acetone before being air dried.

### Simulated splash assay of AMS efficacy

**Fig. 1.**
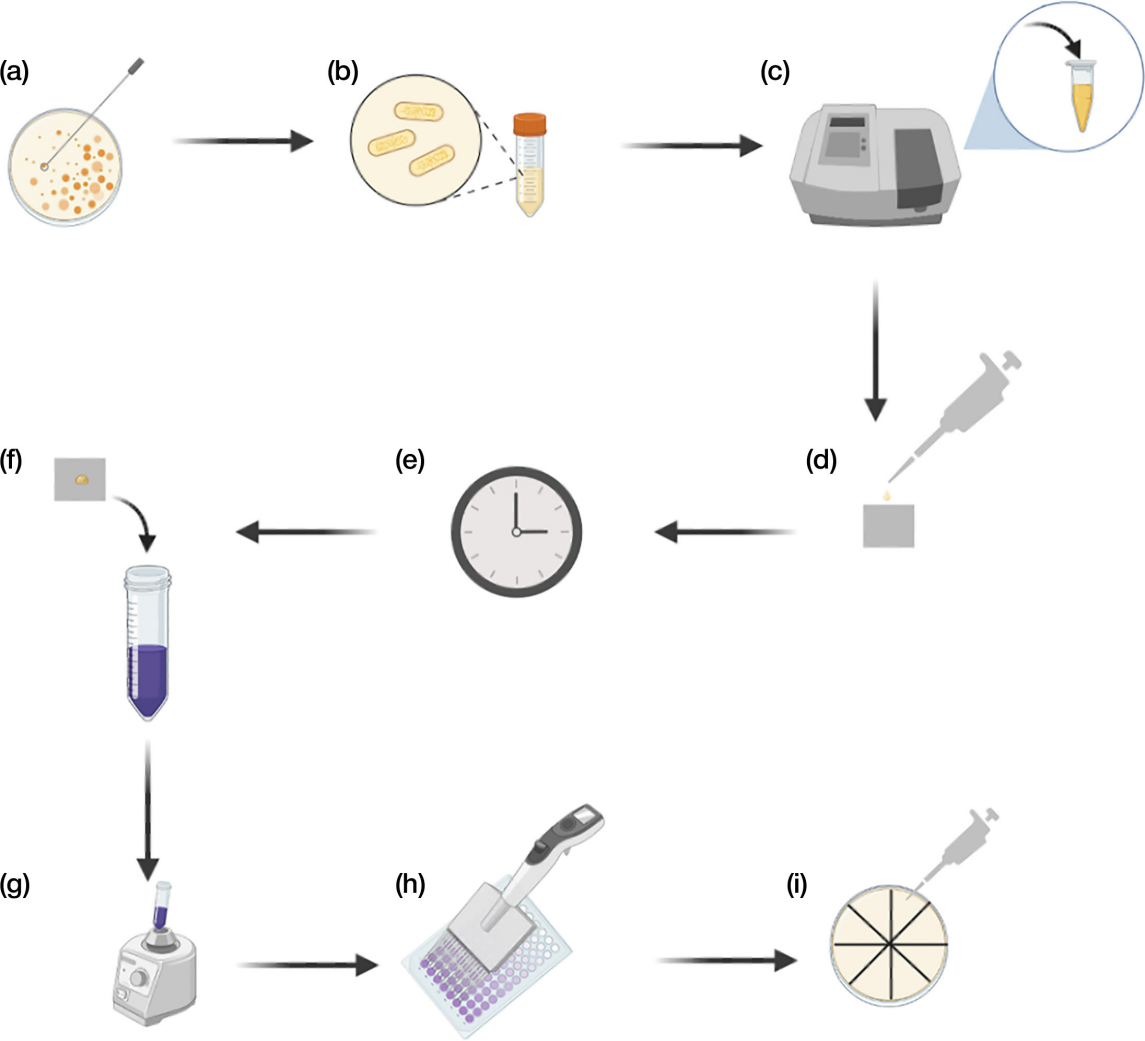
Schematic of the antimicrobial efficacy testing method. (**a**) Bacterial colonies of interest are chosen and (b) grown in LB overnight at 37 °C for 18 h. (**c**) Bacterial culture is adjusted to ~1×10^9^ c.f.u. ml^−1^. (**d**) The normalized bacteria are pipetted onto steel surfaces and (e) incubated at room temperature for 30 min. (**f**) Bacteria were recovered by (g) vortex mixing and by (h) serial dilution and (i) pipetted onto agar plates which were then assessed by counting the c.f.u. after ~18-h incubation at 37 °C.

All clinical isolates and WT strains were cultured in 5 ml LB overnight at 37 °C for ∼ 18 h with shaking (180 r.p.m.) before being adjusted to ∼1×10^9^ c.f.u. ml^−1^. The CHX-coated antimicrobial steel and stainless steel control were placed in a 12-well tissue culture plate (Corning). Cultures were pipetted onto test surfaces as 9×1 µl drops in a simulated splash test (*∼*1×10^6^ cells per 1 µl culture). This was followed by a 30-min incubation of the surface at room temperature (18–22 °C). These incubation periods were chosen for comparative analysis against previously reported studies that concluded successful antimicrobial efficacy of CHX after 30 min when attached to nitrided acrylonitrile butadiene styrene-coated surfaces [31]. Bacteria were recovered from the surfaces by vortex mixing for 1 min in 10 ml sterile Dey-Engley neutralizing broth with seven to ten zirconium oxide beads. The neutralizing broth suspension was then serially diluted in sterile PBS, and survival was assessed by evaluating c.f.u. after ∼18-h incubation at 37 °C on LB agar. The c.f.u. in the original suspension was also analysed by dilution, incubation on LB agar and c.f.u. counting as for the test surfaces (Fig. 1). Tests were carried out three times over separate days to produce a total of nine sets of repeats for each clinical isolate. Statistical analysis was performed using SPSS Statistics software packages (version 28.0.1.1; IBM). Data were analysed using an independent sample t-test, and *α* was set to 0.05.

### MIC assays in solid and liquid media

To determine the MIC for CHX in solid LB agar, all strains were tested against various CHX concentrations. For solid surface MIC tests, strains were inoculated using a BM6-BC robot (S and P Robotic Inc.) onto 2% agar LB petri dishes containing different concentrations of CHX ranging from 0.1 to 80 µM. All inoculated plates were incubated for 12–14 h at 37 °C and then imaged using controlled lighting with an 18-megapixel Canon Rebel T3i (Canon) on the BM6-BC robot (S and P Robotic Inc.). The resulting images were then quantified using the image analysis software Iris [35]. Fitness across mutants was calculated based on the normalized density values where the differences amongst conditions in the median and sd were removed by calculating a colony size density z-score per image [35].

The MIC for CHX in liquid media was determined by broth microdilution assay as described previously [36]. Briefly, bacteria were incubated in 5 ml of LB with shaking for ~16 h at 37 °C. Cultures were diluted 1/2,000 in LB before being used to inoculate dilution series of CHX in LB in a 96-well plate before being incubated at 37 °C for ~18 h. The MIC was recorded as the lowest concentration that inhibited growth, and experiments were repeated in triplicate.

## Results

To test the effectiveness of CHX on bacterial fitness, 91 clinical isolates from a diverse range of bacteria were obtained from the QEH Birmingham, UK (Table 1). The isolates were identified using matrix-assisted laser desorption/ionization-time of flight MS validation by QEH Birmingham and comprise both Gram-positive and Gram-negative bacteria [37]. Commonly used laboratory strains of *E. coli* (BW25113 and ATCC25922), *P. aeruginosa* (PA01) and *Staphylococcus aureus* (ATCC6538) were also included for comparison. The ability and efficiency of CHX to act as an antimicrobial agent towards these isolates when coated on steel surfaces were examined.

**Table 1.** The clinical isolate samples obtained from the QEH Birmingham, UK

Type of species	Species name	No. of strains tested
Gram-positive	Methicillin-sensitive *S. aureus*	5
	Methicillin-resistant *S. aureus*	8
	*Enterococcus*	2
	Coagulase-negative *S. aureus*	6
	*Bacillus*	2
	*Staphylococcus epidermidis*	2
	*S. aureus*	1
Gram-negative	*P. aeruginosa*	12
	*Klebsiella pneumoniae*	11
	*Enterobacter cloacae*	14
	*E. coli*	13
	*Moraxella catarrhalis*	4
	*Citrobacter*	1
	*Haemophilus influenzae*	5
Unknown	–	5

Each isolate was labelled with a unique identifier for screening purposes. Details of all isolates and the assigned identifiers are included in Table S1 (available in the online Supplementary Material). To further study the antimicrobial effects of CHX, the MIC of each isolate was then measured as a control in both solid and liquid LB. As such, the resistance of each isolate to CHX could be compared across different modes of exposure to determine whether any observed antimicrobial resistance is limited to any one mode of exposure, such as CHX-coated steel.

### Antimicrobial efficacy of CHX-coated nitrided steel surfaces

We have previously demonstrated the efficacy of our AMS steel surfaces against model laboratory strains of bacteria and have shown that the CHX-coated polymer surfaces are active against laboratory strains of *E. coli* and *S. aureus* that had been evolved for resistance to CHX in liquid media [27,31]. Whether this observation was also true for clinical isolates that are resistant to CHX in solution is unknown, as these organisms could have more diverse genetic backgrounds and potential resistance mechanisms. Therefore, we aimed to assess the efficacy of the surfaces against 91 environmental/clinical isolates sourced from QEH Birmingham.

**Fig. 2.**
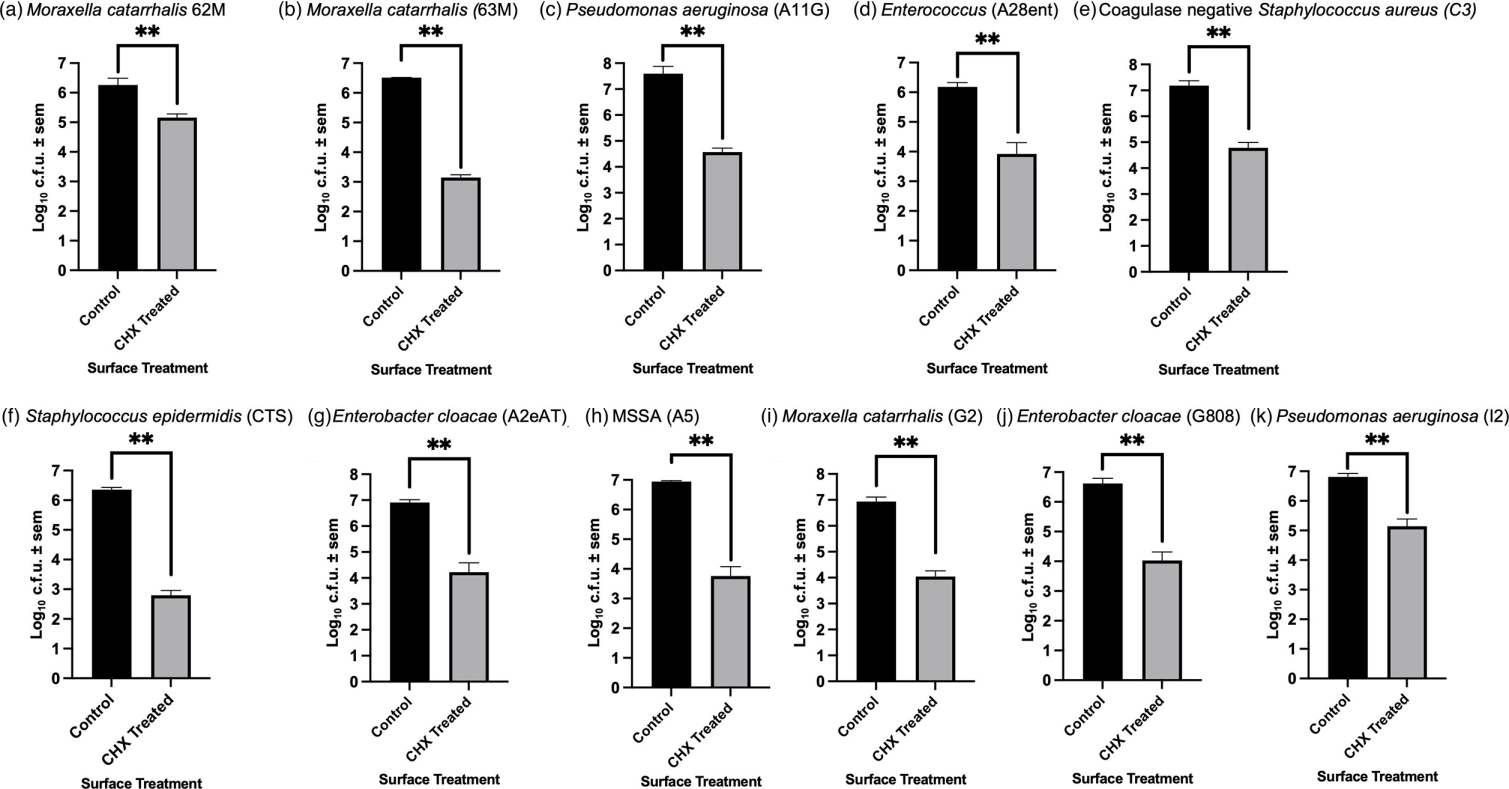
CHX-resistant isolates (labelled) and their respective controls. (**a**) *Moraxella catarrhalis* (62M), (**b**) *M. catarrhalis* (63M), (**c**) *P. aeruginosa* (A11G), (**d**) *Enterococcus* (A28ent), (**e**) Coagulase-negative *S. aureus* (C3), (**f**) *Staphylococcus epidermidis* (CTS), (**g**) *Enterobacter cloacae* (A2eAT), (**h**) Methicillin-sensitive *S. aureus* (MSSA) (A5), (**i**) *Moraxella catarrhalis* (G2), (**j**) *Enterobacter cloacae* (CRE) (G808) and (**k**) *P. aeruginosa* (I2). Bacterial quantification of all 11 isolates that were resistant to CHX-coated surfaces, *n*=3, error bars showing sd. All isolates were shown to be resistant across all three sets of repeated experiments. Statistical analysis confirmed that all isolates had a statistical significance <0.001 after normal distribution.

Nitrided steel surfaces were coated with CHX by incubation with peptide coupling agents as previously described [33]. Each isolate was exposed to the surfaces in simulated splash tests over a period of 30 min followed by serial dilution and incubation at 37 °C for ∼18 h. The 30-min incubation period was determined as appropriate based on previous experiments with AMS steel and the CHX-coated polymer surfaces [27,31, 33]. After 30-min incubation periods, over 85% of the clinical isolates tested (80 clinical isolates) displayed significant differences between the control (stainless steel) and CHX-coated surfaces (7–8 log reductions in survival) (Figs S1–S3). The 11 remaining clinical isolates showed some resistance to CHX with 3–4 log reductions compared with the control (Fig. 2), as opposed to the large reductions in survival observed for the rest of the isolates. In both the sensitive and weakly resistant groups, three sets of repeated experiments were completed, and the results were consistent across these tests.

Statistical analyses were performed using SPSS Statistics software packages (version 28.0.1.1; IBM), and the data of all 11 isolates were analysed via an independent sample t-test. Recorded *P*-values were <0.001 for all isolates, denoting that the difference between the control samples (without CHX surface attachment) and samples with CHX surface attachment was still significant at a 3–4 log reduction. Similarly, the other isolates all had *P*-values <0.001 using the same statistical analyses at greater log reductions (>5 log reductions) (Fig. S4). A typical representation of the dilution series for c.f.u. enumeration is shown in Fig. S5. No clinical isolate that had been exposed to CHX after 30 min displayed identical log values as the control, indicating that whilst some strains showed weak resistance, none were entirely unaffected by the AMSs. Amongst the 11 bacteria that were resistant to the surfaces, we found that 7 were Gram-negative species (3 *Moraxella catarrhalis*, 2 *P*. *aeruginosa* and 2 *Enterobacter cloacae*) and 4 were Gram-positive (1 *Staphylococcus epidermidis*, 1 coagulase-negative *S. aureus*, 1 *Enterococcus* and 1 methicillin-sensitive *S. aureus*). Whilst this was a relatively small number of isolates identified as being resistant to the surfaces, there appeared to be no particularly strong bias based on species or Gram status (Table 2). One isolate *of S*. aureus and two *P*. *aeruginosa* were also partially resistant in two of the three tests (Figs S6 and S7). Non-resistant strains and isolates are also shown in Figs S1–S3.

**Table 2.** Table displaying the different types of bacteria that were tested and the number of those that are resistant to CHX across all three modes of CHX integration

	Bacterial strain	No. of strains tested	No. of resistant isolates		
			AMS	Solid LB	Liquid LB
**Gram-positive**	Methicillin-sensitive *S. aureus*	5	1	–	1
	Methicillin-resistant *S. aureus*	8	–	3	1
	*Enterococcus*	2	1	1	1
	Coagulase-negative *S. aureus*	6	1	–	3
	*Bacillus*	2	–	–	1
	*S. epidermidis*	2	1	–	1
	*S. aureus*	1	–	–	–
**Gram-negative**	*P. aeruginosa*	12	2	1	–
	*Klebsiella pneumoniae*	11	–	4	5
	*E. cloacae*	14	2	4	6
	*E. coli*	13	–	1	1
	*M. catarrhalis*	4	3	–	–
	*Citrobacter*	1	–	–	–
	*Haemophilus influenzae*	5	–	–	–
**Unknown**	Unknown	5	–	2	1

Resistance on liquid LB was categorized as growth at ≥5 µM or more, on solid media as ≥60 µM or more and for the antimicrobial steel as demonstrating any detectable growth after exposure.

### MIC of CHX against clinical isolates

**Fig. 3.**
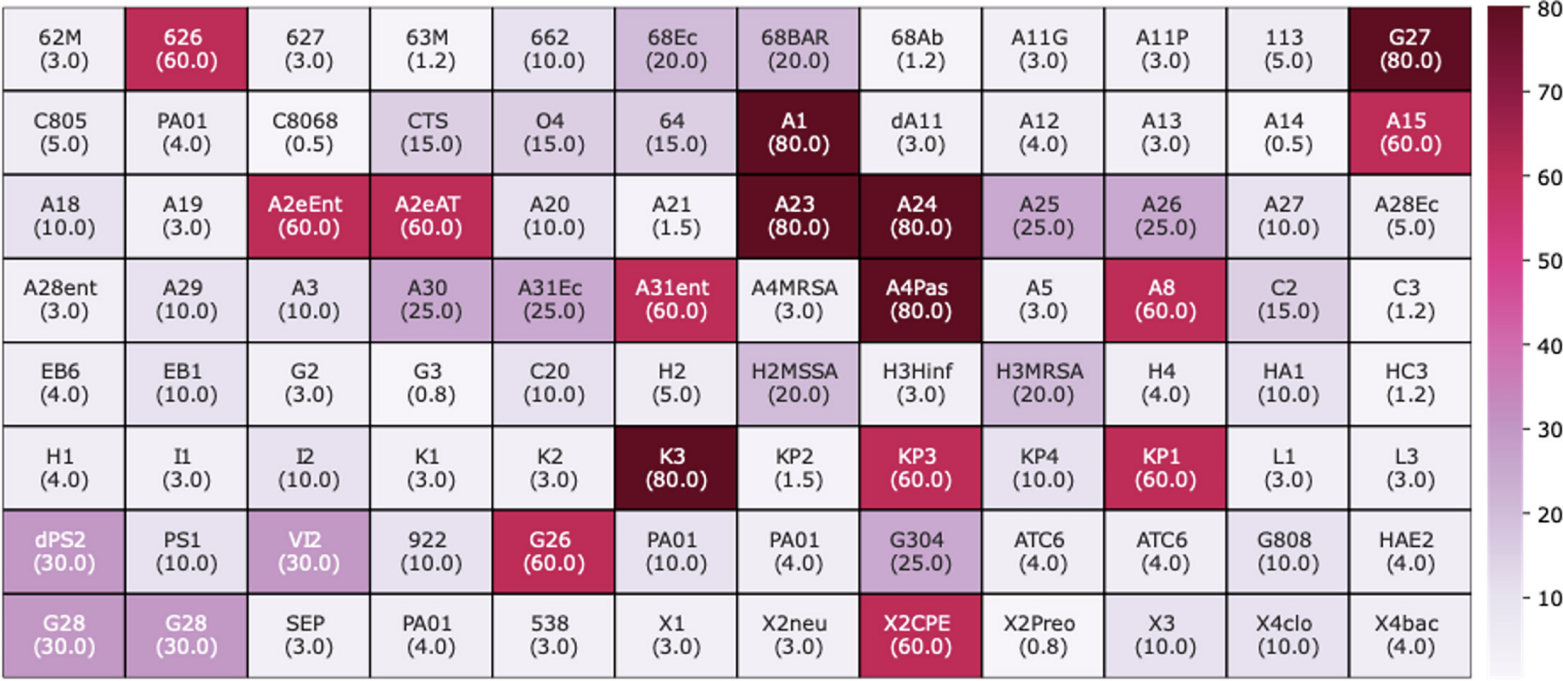
Clustered heatmaps displaying MIC values for the clinical isolates assayed against CHX dissolved in solid LB medium. CHX concentrations in LB agar plates varied from 0.1 to 80 μM (highlighted underneath each isolate's unique identifier and by the scale bar).

Having established that the CHX-coated surfaces are effective against 85% of the clinical isolates tested, we thought to correlate resistance to the AMS with the capacity to resist CHX in the more common formulation of being dissolved in liquid. Therefore, we sought to quantify the level of resistance to CHX that was freely dissolved in either solid agar or in liquid broth, as this could potentially change the susceptibility. Clinical isolates were arranged in 96-well plates to facilitate high-throughput MIC assays, and this array was kept consistent throughout MIC assays in both solid and liquid LB. For the solid media MIC, strains were pinned onto LB agar supplemented with varying concentrations of CHX and incubated for 18 h at 37 °C before being imaged and analysed using IRIS image analysis software [35]. Fitness was established based on colony growth at specific concentrations. MIC values were recorded at concentrations in which no growth was observed. These MIC results were then plotted as a heatmap for all isolates tested (Fig. 3).

There were a greater number of isolates that were resistant to CHX on solid LB compared with CHX-coated nitrided steel, as we found 16 isolates that were resistant to the highest 2 concentrations of CHX tested (80 and 60 µM). Surprisingly, only one of the isolates that was resistant to the surfaces was also found to be resistant to CHX when dissolved in solid LB agar: A2eAT, a strain of *E. cloacae*. We also found that two other *E. cloacae* strains were resistant to the same level of CHX (60 µM) dissolved in solid LB agar as A2eAT (A31ent and A2eEnt), despite not being resistant to the AMS, suggesting the potential for alternative mechanisms of resistance between the strains.

**Fig. 4.**
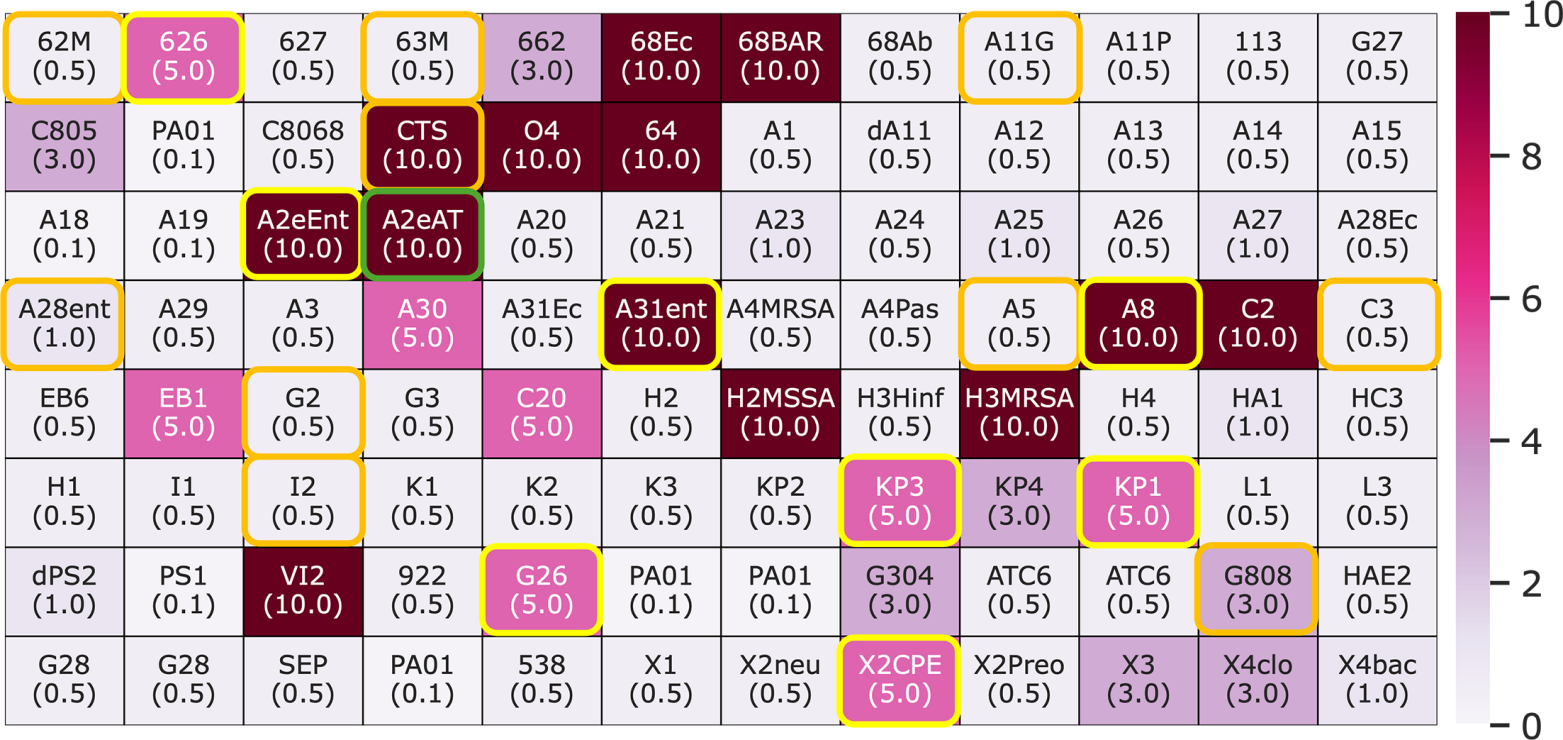
Clustered heatmaps displaying the MIC values for the clinical isolates with CHX in liquid LB medium. CHX concentrations on LB plates varied from 0.1 to 10 µM, and the values of the MIC for each clinical isolate are highlighted. The isolates that were resistant to CHX in both solid and liquid LB are highlighted by the yellow circles, isolates resistant on the nitrided steel surfaces are highlighted by the orange circles and the only isolate that was found to be resistant to CHX across all three surface modalities is highlighted by the green circle.

To further study the effects of increased levels of diffusion on the antimicrobial efficacy of CHX, we then tested the MIC of CHX dissolved in liquid LB. This enabled us to investigate the effect of diffusion ranging from no levels of diffusion (CHX-coated nitrided steel) to very high levels of diffusion (CHX in liquid LB). A similar heatmap in the same format was generated for the MIC for each clinical isolate when grown in liquid LB with CHX (Fig. 4). We found 21 isolates that were resistant to the highest 2 concentrations of CHX tested (10 and 5 µM), which is the most of all three modes of CHX activity tested. The resistant strains were not species specific and included methicillin-sensitive *S. aureus*,methicillin resistant *S. aureus*, *Bacillus*, *Klebsiella pneumoniae*, *E. coli*, *Enterococcus*, *E. cloacae*, *S. epidermidis* and coagulase-negative *Staphylococcus* species. Of the strains resistant to 5 µM CHX, six of the eight strains were *K. pneumoniae*, whereas amongst the strains resistant to 10 µM, there was more variety despite being mostly dominated by *E. cloacae* and *Staphylococcus* species. All AMS-resistant strains were also not resistant to CHX in liquid media, except for a strain of *S. epidermidis* with the identifier CTS and A2eAT, which was resistant to CHX in all three modes of exposure. Unexpectedly, we also found that the MIC to CHX in solid agar did not predict resistance to CHX in liquid broth.

**Fig. 5.**
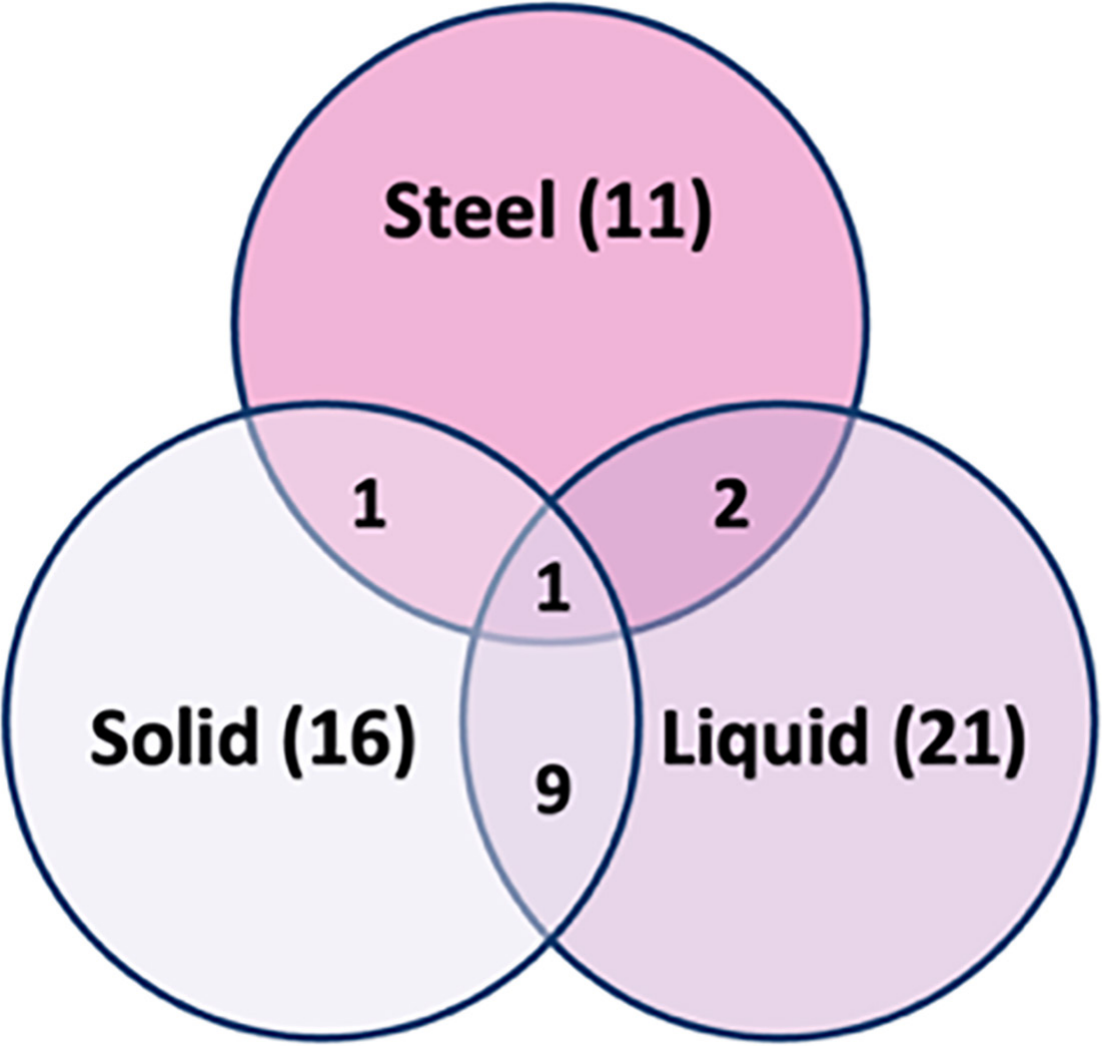
Venn diagram comparing resistant strains between all three modes of CHX action (coated on steel surfaces and coated on a solid LB surface and in liquid LB). Values in brackets show the number of CHX-resistant strains in each particular mode of CHX exposure, and values in overlapping circles depict the strains that show CHX resistance in two or more methods. Resistance on steel was selected for CHX when strains were resistant across all three sets of tests. On solid and liquid media, resistance was selected when MIC value ≥60 µM (solid media) and an MIC value ≥5 µM (liquid media).

From the data generated across all three modes of CHX integration, a Venn diagram was produced to show the relationships in the resistance of the clinical isolates across all three modes of CHX exposure (Fig. 5). The data indicate that most of the isolates were found to be resistant to CHX when integrated in liquid LB (21 isolates) followed by solid LB (16 isolates) and steel (11 isolates). Resistance was defined when no growth was observed at MIC values ≥60 µM (solid) and ≥5 µM (liquid). There was only a single strain of the *E. cloacae* species that was resistant across all three modes of CHX exposure. Decreasing our threshold for classification as resistant to ≥30 μM for solid agar, ≥3 μM for broth and strains that show resistance in 2 of the 3 surface tests results in 14 resistant strains for steel, 19 for solid and 28 for liquid. Despite this, the data are still not predicative of strain resistance between modes of exposure, and overlaps in resistance only increase by one between solid and liquid and one between steel and liquid, but no increase in the number of resistant strains across all three modes. We then attempted to compare the bacterial species resistance or susceptibility to CHX to see if it was possible to identify whether particular species were resistant in each test (Table 2). However, due to the wide variety in the number of isolates for each species, no formal statistical analysis on the data could be undertaken. Some of the isolates that were tested were also not identified and therefore could not be categorized.

## Discussion

The concept of coating nitrided steel with CHX is an established technique that we have previously developed as an effective AMS technology against model laboratory strains of bacteria, fungi and viruses [27,33]. We also previously tested the CHX-coated polymer surfaces against a strain that had been evolved for resistance to CHX in liquid media under laboratory conditions [31]. As such, we sought to investigate whether organisms that had been isolated from the hospital environment might have resistance to the surfaces, indicating potential mechanisms other than those that are possible through laboratory-based evolution in the background of a single strain. The limitation of investigating a single strain is that the results are confined only to the genetic material the strain had initially at the beginning of the lab-based evolution experiment, whereas the environmental isolates investigated in this research would likely have a much wider genetic diversity.

The antimicrobial efficacy of CHX across different modes of exposure was compared and included the application of CHX on nitrided steel, in solid LB and dissolved in liquid LB. The nitrided steel coated with CHX displayed robust antimicrobial efficacy, with all clinical isolates demonstrating some level of reduction in survival and over 85% being killed after a period of 30 min of being exposed to CHX on these surfaces. In contrast, 79% and 77% of isolates were not resistant to the highest concentrations of CHX incorporated into solid and liquid media, respectively. The highest number of strains that overlapped in resistance to CHX across different surface modalities was found to be between that of the solid and liquid LB. The clinical isolates responsible for these overlaps were from a varied selection of species, and there were no clear trends amongst the same bacterial species. This observation coincides with the concept that such antimicrobial killing factors are complicated as different species of strains will respond subjectively to CHX. This further emphasizes the importance of there being no ‘gold standard’ as a mode of antiseptic eradication of bacterial pathogens in the clinical environment, as bacterial species are fundamentally diverse. Factors such as environmental variability in clinical settings represent limitations within the study, and therefore, the adoption of multiple antimicrobial treatment protocols should be considered.

The patterns of resistance amongst the strains to CHX in agar and broth might have been expected to be similar but to different levels. However, not only were the patterns of resistance to CHX-coated surfaces different to the other modes, but resistance on solid media was not predictive of resistance to CHX in liquid media. This suggests that there are differences in the functional mechanistic action of CHX on solid surfaces compared with liquid. This is surprising, as in both solid agar and liquid broth formulations, the CHX is free to diffuse and therefore penetrate the bacterial cells. However, in the case of the steel surfaces, the CHX is permanently bonded to the surfaces themselves with no detectable leaching over much longer time periods than those used here [33]. As such, the steel-bonded CHX cannot enter the cells, therefore being theoretically limited to interactions with the bacterial cell surface. This potentially keeps the active principal of CHX in contact with the micro-organisms for a longer time, thus inhibiting their growth. Therefore, it might be expected that this would be different to the exposure in liquid and solid media. This is further supported by previous studies, which have shown that the release of CHX is prolonged via a decrease in CHX solubility, as is the case of its precipitation on solid surfaces which fundamentally accomplishes a sustained release of CHX, thus maintaining its killing capability [24]. This observation of strong antimicrobial activity on solid surfaces has also been discussed in other studies and in instances where CHX has been integrated into gel formulations and then compared with that when present in liquid [38]. However, as the CHX in these formulations is ultimately free to diffuse, it provides the potential for resistance to be achieved through a wider variety of resistance mechanisms, such as expression of efflux pumps.

It has been demonstrated through previous studies that major facilitator superfamily (MFS) efflux pumps are crucial for bacterial cells to withstand biocides like CHX as they can actively remove the antimicrobial agent from the bacterial cell [39]. Target alteration or overexpression, efflux pump activation and other processes contribute to the reduced sensitivity of bacteria to biocides [40]. There are many efflux pumps in bacteria, and some of them have been directly linked as contributing towards the decreased susceptibility of bacterial species towards CHX [41,42]. Previous data from other clinical isolates of *E. cloacae* have linked SmvR-like regulators coding for TetR-type transcriptional repressors to having important roles in CHX resistance via repressing the expression of *smvA* (which encodes for an MFS family efflux pump) [39]. Such observations may account for the resistance to CHX in the *E. cloacae* isolate in this research; however, more research would be needed to find out if this were to be the case. Moreover, whilst efflux pumps may be effective in scenarios where CHX can freely diffuse, they may not be as effective on the antimicrobial steel surfaces used here where CHX is not released. Despite CHX still being free to diffuse from the solid agar tested here, localized regions of high CHX concentrations could still occur, therefore potentially requiring alternative mechanisms to overcome the stress of CHX exposure.

Previous studies have identified several additional mechanisms of bacterial resistance to CHX, including alterations in cell membrane permeability, biofilm formation, enzymatic degradation and changes in gene regulation [43]. In addition to alterations in the efflux pumps discussed previously, both cell membrane modifications and enzymatic degradation within bacteria would need to be more robust and immediate in liquid LB medium due to the uniform and constant exposure to CHX. In solid medium, localized adaptations within the broader bacterial population, such as biofilm formation or differential gene regulation, become more pronounced. However, when exposed to the steel-bonded CHX, changes to cell permeability may not be effective, whereas enzymatic degradation could still provide resistance. Genome sequencing of strains of interest, including the *E. cloacae* isolate (A2eAT), which exhibited resistance against all three modes of CHX surface modalities, could have provided interesting information. However, due to the broad range of species from the collection of clinical isolates used here, to truly understand the mechanism of resistance to CHX would require large-scale sequencing and analysis on many isolates of the same species. Such studies would allow further investigations into the genetic profiles of certain strains and their relation to CHX resistance including the functionality of CHX, its mode of action and the associated regulatory pathways involved. One of the key challenges with these experiments however would be studying the genetics in clinical and environmental isolates, which are notoriously difficult to genetically manipulate [44].

Future research in the applications of AMS coatings, including those related to CHX, would focus on expanding the research field across various industries whilst further improving on efficacy, durability and sustainability. Research should focus on developing advanced materials, such as nanostructured coatings, biomimetic surfaces and smart antimicrobial technologies that can dynamically respond to environmental triggers [45]. CHX-based coatings represent a promising direction in AMS technology, particularly in healthcare settings where infection control is critical. Future research should explore improved delivery systems, such as nanoencapsulation [46] and polymer-based controlled-release mechanisms [47] to enhance the sustained efficacy of CHX whilst reducing microbial resistance. Investigating synergistic formulations that combine CHX with other antimicrobial agents or physical surface modifications could further improve antimicrobial performance [48].

Standardization and regulatory considerations will also be essential to ensure the widespread adoption of AMSs, including those incorporating CHX. Furthermore, hospital infection control policies must be able to adapt to these practices by evaluating the long-term effectiveness of these surfaces, updating any cleaning protocols whilst ensuring staff training to maximize the impact of these AMS coatings in reducing HAIs. Interdisciplinary collaborations between material scientists, microbiologists and engineers will drive innovations that balance effectiveness, safety and real-world applicability [49]. By advancing these research directions, AMSs including CHX coatings can adopt a transformative role in improving hygiene, infection prevention and public health across multiple industries.

## Conclusions

We have assessed the antibacterial efficacy of CHX across various modes of integration, targeting a diverse range of Gram-positive and Gram-negative bacterial clinical isolates from the QEH in Birmingham, UK. The most effective mode of CHX exposure against bacterial survival was when permanently bonded to nitrided steel, followed by CHX dissolved within solid and liquid LB media. We have shown that CHX has an impact on all bacterial strains when bonded to nitrided steel after 30 min of incubation at room temperature. The results from these studies will aid in the future research of CHX functionality against various pathogenic bacteria and enable tailored application of CHX to be implemented within clinical settings. In conclusion, the results highlight the promising potential of CHX AMS coatings for widespread adoption in healthcare settings. These coatings provide effective protection against a wide range of bacterial pathogens, playing a crucial role in preventing the spread of HAIs. Whilst the technology is a relatively simple and cost-effective modification of current stainless steel designs for existing hospital surfaces, large-scale implementation would require the replacement or off-site modification of existing materials.

## Supplementary material

10.1099/jmm.0.002025 Uncited Supplementary Material 1.

10.1099/jmm.0.002025 Uncited Table S1.

## Abbreviations

AMS: antimicrobial surface
CHX: chlorhexidine digluconate
HAIs: healthcare-associated infections
LB: lysogeny broth
MFS: major facilitator superfamily
QEH: Queen Elizabeth Hospital
