# Supplementary material for: Antimicrobial efficacy of chlorhexidine-treated surfaces against clinical isolates implicated in nosocomial infections
Source: J Med Microbiol. 2025 Jun 24;74(6):002025. doi: 10.1099/jmm.0.002025 (PMC12188002; doi:10.1099/jmm.0.002025)

Antimicrobial efficacy of chlorhexidine treated surfaces against clinical isolates implicated in nosocomial infections

SUPPLEMENTARY RESULTS

S1 CHX non-resistant Gram-positive isolates to CHX on steel surfaces

A) Methicillin Resistant *Staphylococcus aureus*

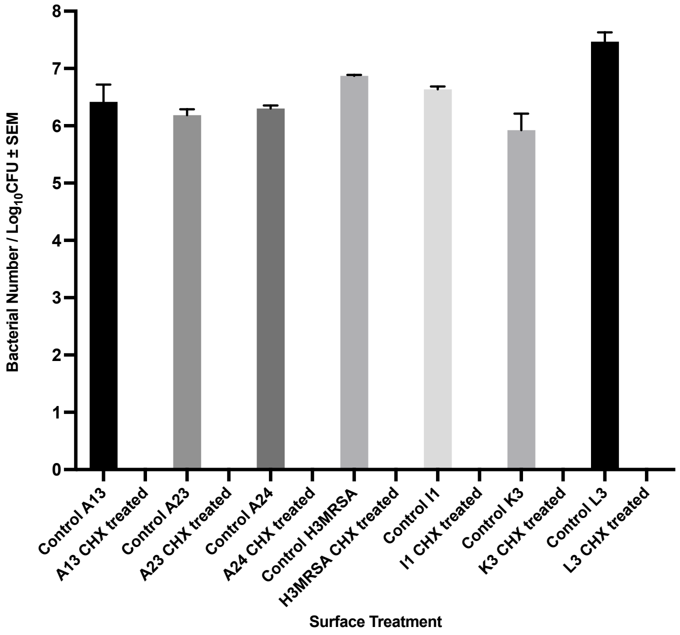

B) Methicillin Sensitive *Staphylococcus aureus*

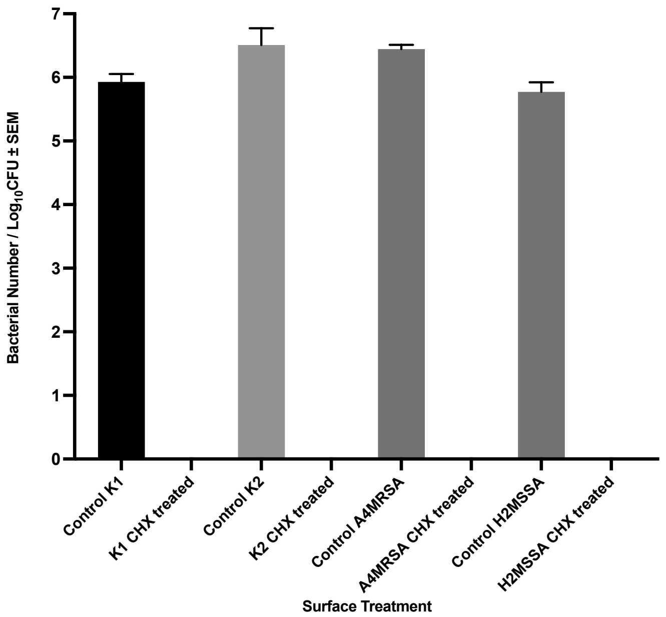

C) ***Staphylococcus aureus***

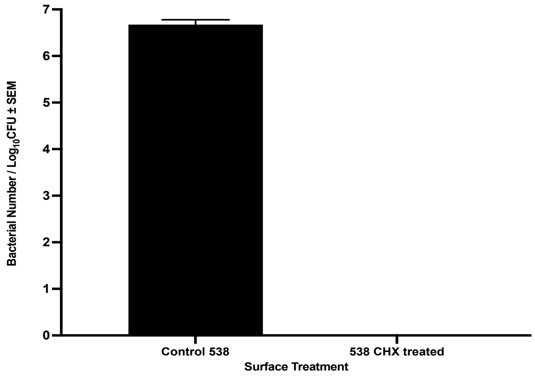

D) **Bacillus**

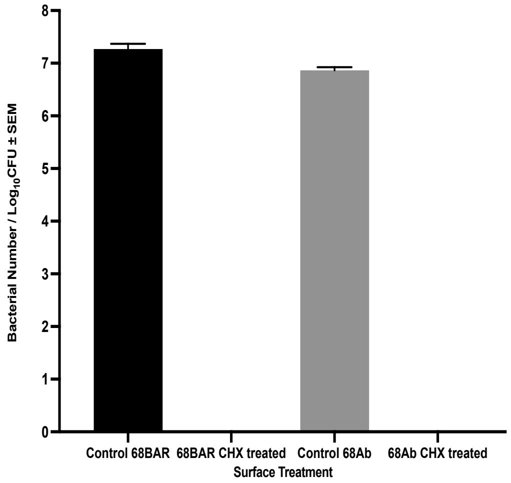

E) ***Staphylococcus epidermidis***

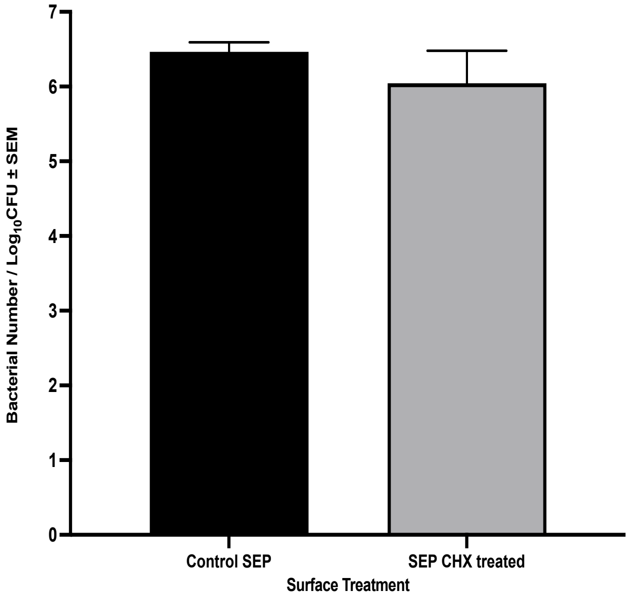

F) **Coagulase negative *Staphylococcus aureus***

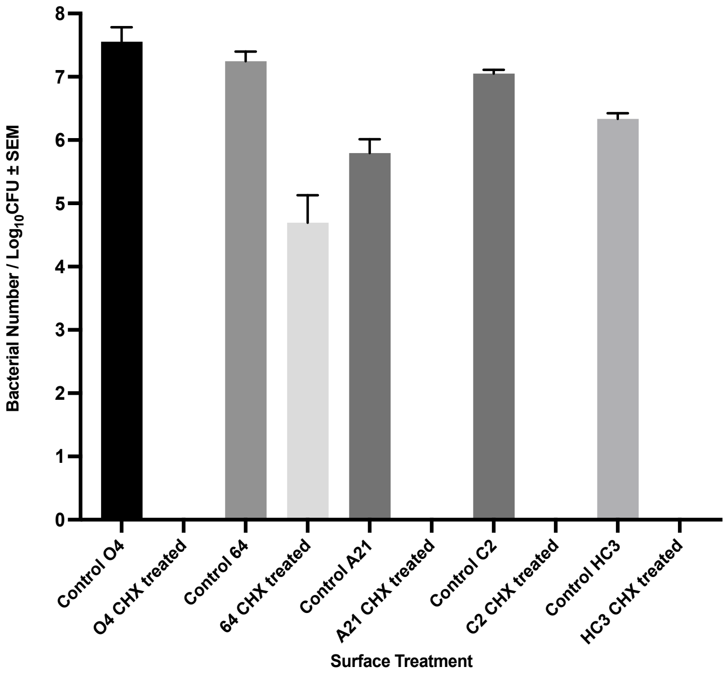

G) Enterococcus

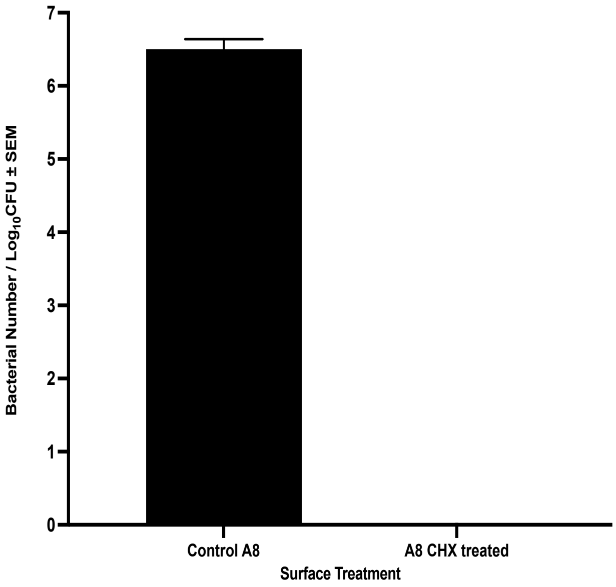

S2 CHX non-resistant Gram-negative isolates to CHX on steel surfaces

A) Escherichia coli

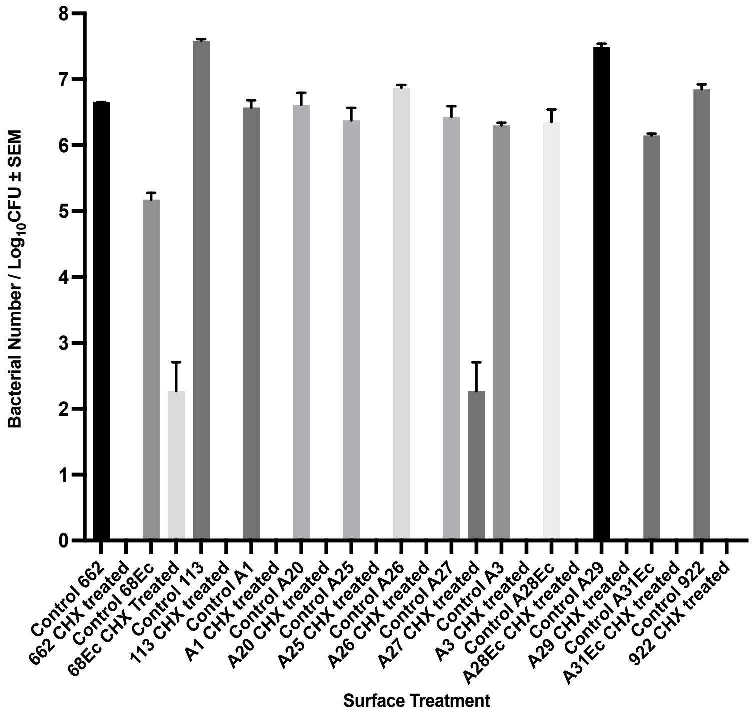

B) Pseudomonas aeruginosa

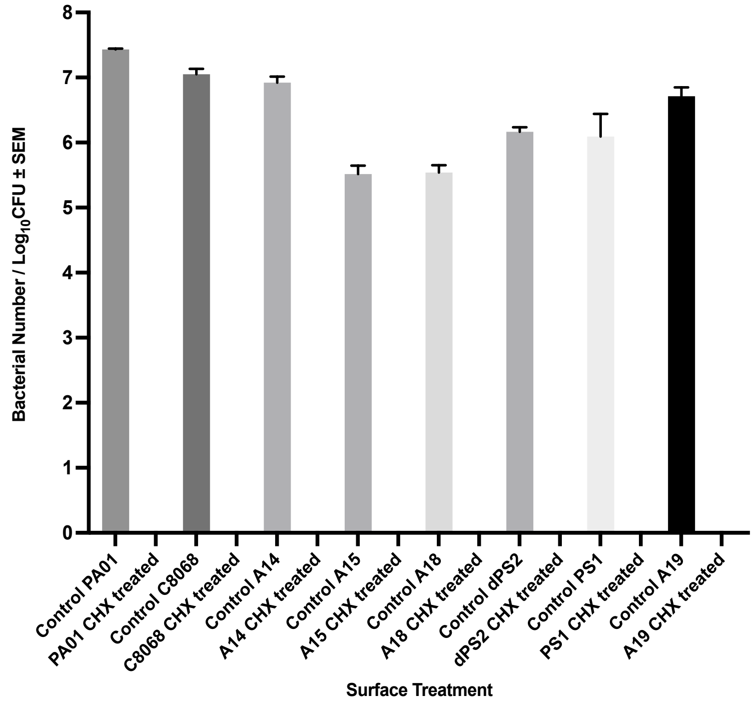

C) ***Klebsiella pneumoniae***

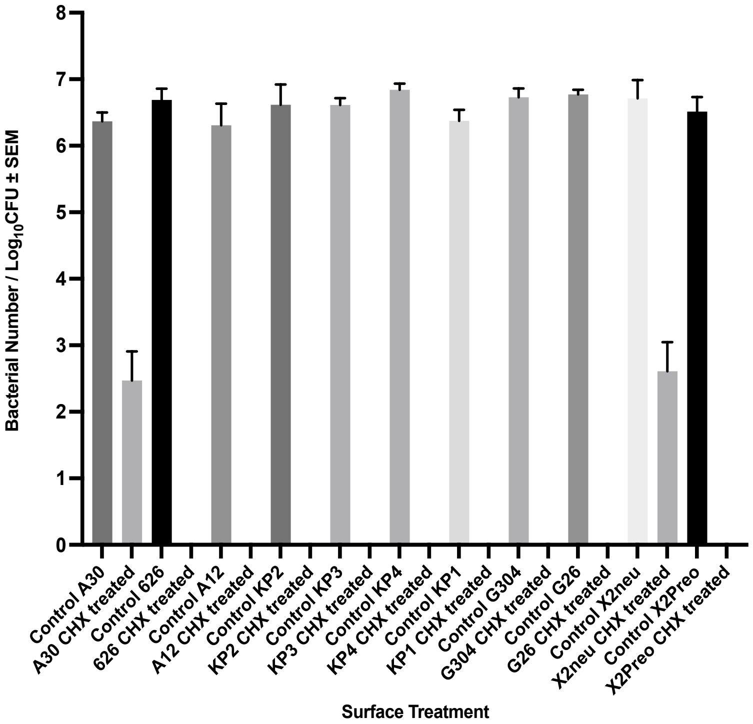

D) ***Moraxella catarrhalis***

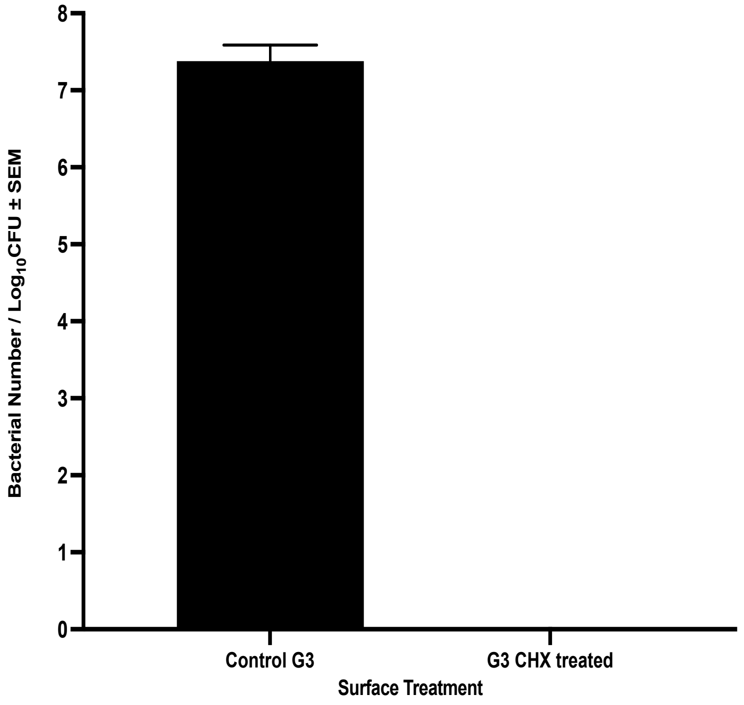

E) Citrobacter

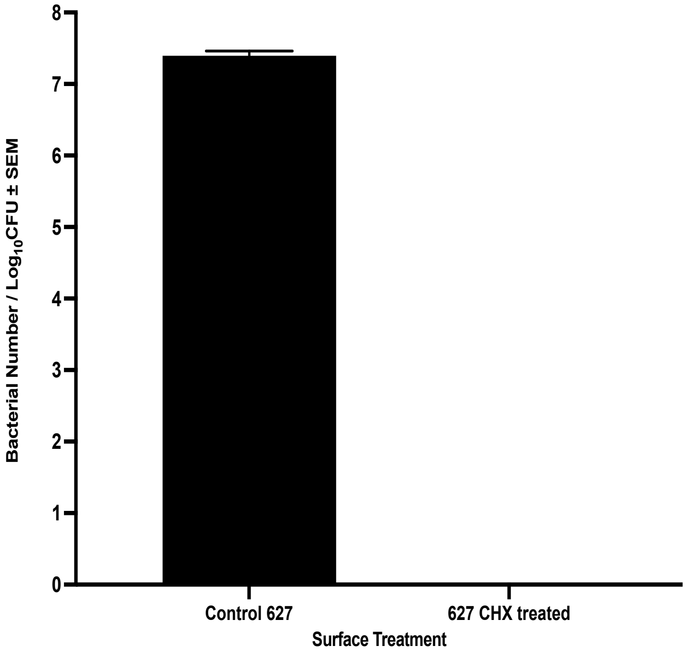

F) Enterobacter cloacae

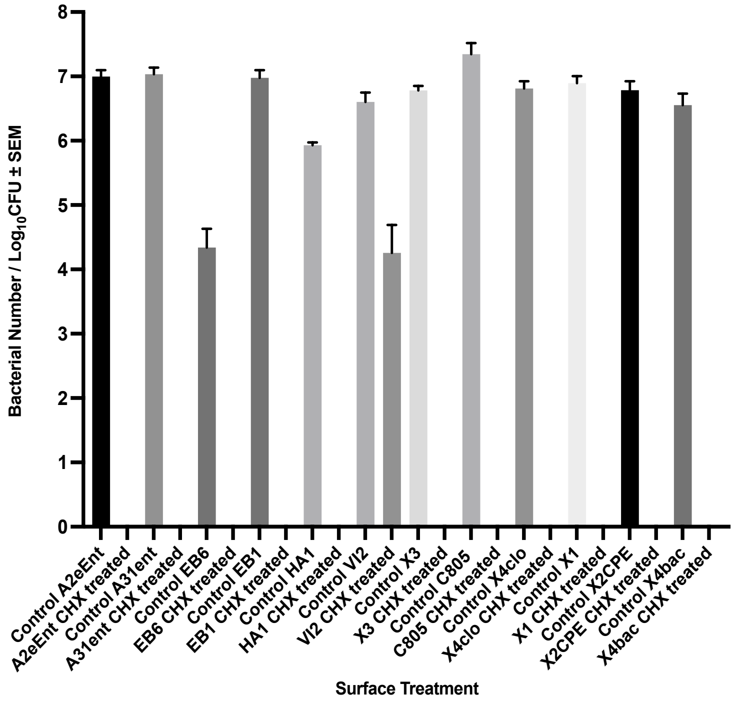

G) Haemophilus influenzae

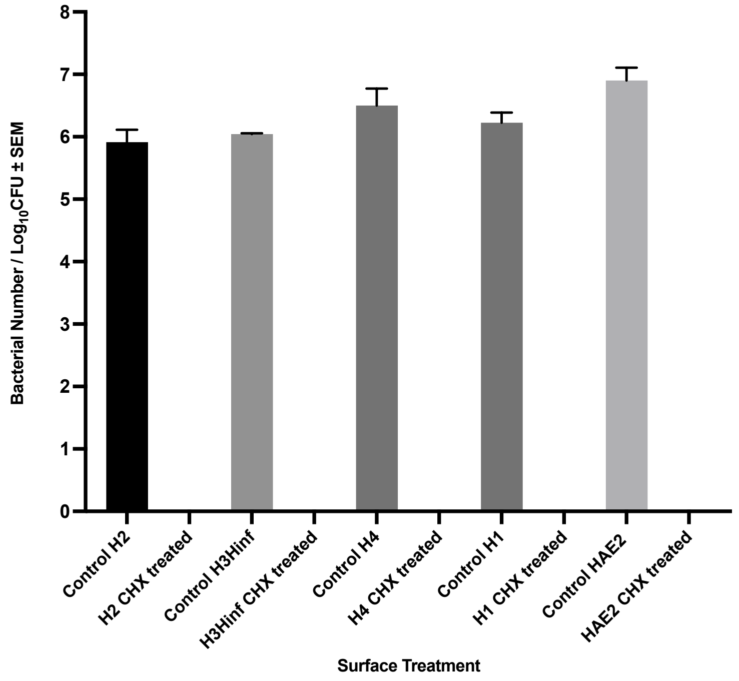

S3 CHX non-resistant unknown isolates

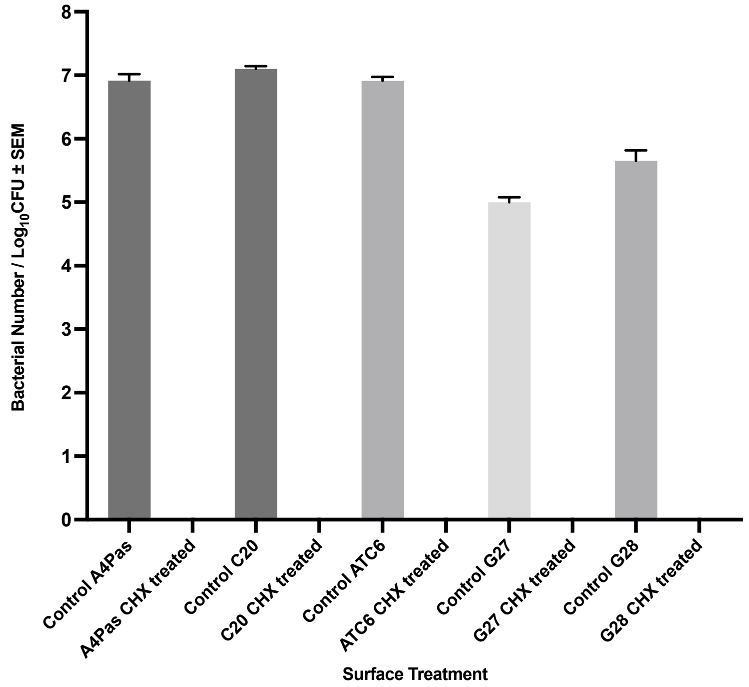

S4 CHX resistant isolates to CHX on steel surfaces statistical analysis

Independent Samples Test

		Levene's Test for Equality of Variances		t-test for Equality of Means						95% Confidence Interval of the Difference	
		F	Sig.	t	df	Significance One-Sided p	Significance Two-Sided p	Mean Difference	Std. Error Difference	Lower	Upper
62M var	Equal variances assumed	0.689	0.419	5.193	16	0.000	0.000	1.19400	0.22990	0.70663	1.68137
	Equal variances not assumed			5.193	11.277	0.000	0.000	1.19400	0.22990	0.68950	1.69850
63M var	Equal variances assumed	0.086	0.773	59.576	16	0.000	0.000	3.37282	0.05661	3.25281	3.49284
	Equal variances not assumed			59.576	14.863	0.000	0.000	3.37282	0.05661	3.25206	3.49359
A11G data	Equal variances assumed	19.130	0.000	3.537	16	0.001	0.003	39610444.44444	11199780.69078	15867970.00794	63352918.88095
	Equal variances not assumed			3.537	8.000	0.004	0.008	39610444.44444	11199780.69078	13783723.71502	65437165.17387
A28ent var	Equal variances assumed	31.654	0.000	5.763	16	0.000	0.000	3.64924	0.63325	2.30681	4.99167
	Equal variances not assumed			5.763	8.216	0.000	0.000	3.64924	0.63325	2.19563	5.10285
C3 var	Equal variances assumed	7.190	0.016	4.582	16	0.000	0.000	3.37276	0.73605	1.81240	4.93313
	Equal variances not assumed			4.582	9.592	0.001	0.001	3.37276	0.73605	1.72323	5.02230
CTS var	Equal variances assumed	262.980	0.000	8.481	16	0.000	0.000	4.60505	0.54298	3.45398	5.75613
	Equal variances not assumed			8.481	8.420	0.000	0.000	4.60505	0.54298	3.36372	5.84639
A2eAT var	Equal variances assumed	8.236	0.011	6.589	16	0.000	0.000	3.94463	0.59869	2.67545	5.21380
	Equal variances not assumed			6.589	8.294	0.000	0.000	3.94463	0.59869	2.57250	5.31675
A5 var	Equal variances assumed	2.556	0.129	9.101	16	0.000	0.000	3.82764	0.42055	2.93610	4.71918
	Equal variances not assumed			9.101	8.500	0.000	0.000	3.82764	0.42055	2.86769	4.78759
G2 var	Equal variances assumed	378.538	0.000	6.012	16	0.000	0.000	4.56284	0.75894	2.95395	6.17172
	Equal variances not assumed			6.012	8.219	0.000	0.000	4.56284	0.75894	2.82079	6.30488
G808 var	Equal variances assumed	94.928	0.000	6.078	16	0.000	0.000	4.34874	0.71554	2.83186	5.86562
	Equal variances not assumed			6.078	8.280	0.000	0.000	4.34874	0.71554	2.70835	5.98913
I2 var	Equal variances assumed	238.633	0.000	4.798	16	0.000	0.000	4.46095	0.92983	2.48980	6.43210
	Equal variances not assumed			4.798	8.045	0.001	0.001	4.46095	0.92983	2.31884	6.60306

S5 LB agar plate representing a typical example of a resistant strain

Comparison of the number of bacterial colonies that are recovered from the control steel surfaces (top) compared to the bacteria recovered from the CHX coated surfaces after 30 minutes of incubation (below). Example is from *E. coli* ATCC25922.

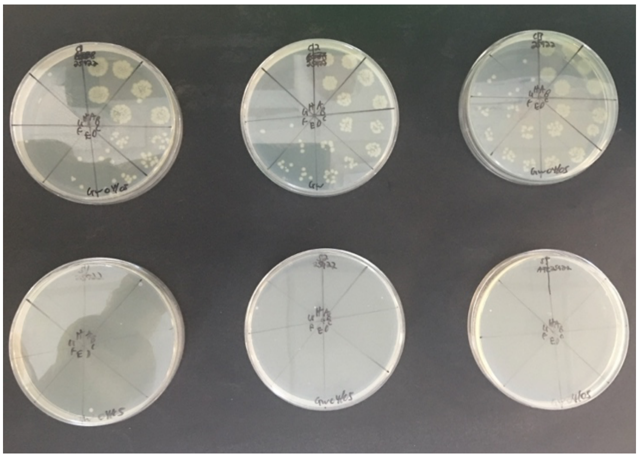

S6 CHX partial resistance Gram-positive isolate to CHX on steel surfaces

A) Methicillin Resistant *Staphylococcus aureus* (L1)

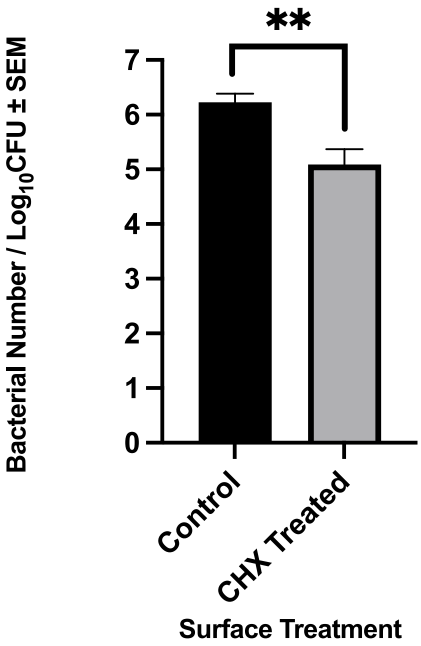

S7 CHX partial resistance Gram-negative isolates to CHX on steel surfaces

A) Pseudomonas aeruginosa

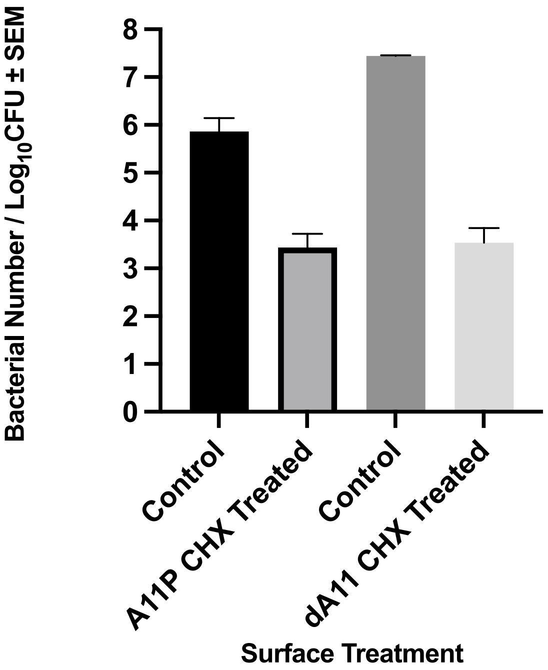

Supplement: Uncited Supplementary Material 1. [file jmm-74-02025-s001.pdf]
